# Evolution and strain diversity advance exploration of *Candida albicans* biology

**DOI:** 10.1128/msphere.00641-23

**Published:** 2024-07-16

**Authors:** Matthew Z. Anderson, Siobhan M. Dietz

**Affiliations:** 1 Department of Medical Genetics, Laboratory of Genetics, University of Wisconsin—Madison, Madison, Wisconsin, USA; 2 Center for Genomic Science Innovation, University of Wisconsin—Madison, Madison, Wisconsin, USA; 3 Cellular and Molecular Pathology Graduate Program, University of Wisconsin—Madison, Madison, Wisconsin, USA; University of Georgia, Athens, Georgia, USA

**Keywords:** *Candida*, aneuploidy, genome variation, genetic diversity, strain diversity, mutation, adaptation

## Abstract

Fungi were some of the earliest organismal systems used to explore mutational processes and its phenotypic consequences on members of a species. Yeasts that cause significant human disease were quickly incorporated into these investigations to define the genetic and phenotypic drivers of virulence. Among *Candida* species, *Candida albicans* has emerged as a model for studying genomic processes of evolution because of its clinical relevance, relatively small genome, and ability to tolerate complex chromosomal changes. Here, we describe major recent findings that used evolution of strains from defined genetic backgrounds to delineate mutational and adaptative processes and include how nascent exploration into naturally occurring variation is contributing to these conceptual frameworks. Ultimately, efforts to discern adaptive mechanisms used by *C. albicans* will continue to divulge new biology and can better inform treatment regimens for the increasing prevalence of fungal disease.

## INTRODUCTION

Adaptation through genetic change affects all biological systems from host-dependent viruses to free-ranging metazoans. Among eukaryotes, fungi have provided an ideal platform for investigating these processes because of their short generation times, ease of manipulation, and shared genomic architecture and regulatory mechanisms with more challenging eukaryotic systems. Among fungi, *Candida albicans* emerged as a model system to study genome evolution due to its significant clinical importance and high level of genome plasticity. *C. albicans* resides as a commensal across multiple highly diverse environments of the human host, including the skin, the oral cavity, the gastrointestinal tract, and the genitourinary tract, with massive shifts in pH, salinity, -oxic conditions, and other environmental factors across these body sites. Mutations in the *C. albicans* genome facilitate adaptation to these commensal niches and can promote opportunistic disease of both its resident mucosal sites and of internal organ systems during invasive disease (1–6). Mechanisms to overcome antifungal drugs also commonly rely on mutation and genomic rearrangements, ultimately leaving certain human populations vulnerable to chronic and acute fungal disease.

The investigation of *C. albicans* genome dynamics by others in the field played an integral part to the personal scientific development of the lead author. The presence of expanded gene families at *C. albicans* subtelomeres first piqued my interest as a prospective postdoctoral researcher when looking to enter the mycology field (7). Immediately prior to and then throughout my postdoctoral training, major advances established genome comparisons among *Candida* species (8, 9), provided some of the first descriptions of intraspecies genetic variation (8, 10), and began to describe the frequency of genomic changes in different *in vitro* and *in vivo* conditions (11–13). This work set the stage to delve deeper into how mutation contributes to adaptive processes.

When writing the mSphere of Influence commentary in 2019 that forms the basis for this Full Circle update (14), two papers were particularly influential in advancing the study of *C. albicans* evolution and adaptation. The first paper described the *in vivo* adaptation of diploid *C. albicans* cells in the oral cavity of mice using the genome reference strain SC5314 (15). Unexpectedly, cells that acquired a trisomy of chromosome 6 (Chr6) from the diploid inoculum displayed increased fitness in this niche. Selection for Chr6 trisomy *in vivo* stood in stark contrast to the expectation that aneuploid cells would be less fit under most conditions compared with their diploid counterparts (12, 16) and suggested that our understanding of mutations that promote host adaptation was incomplete. The second study used whole-genome sequencing of 182 isolates to massively expand the scope of comparative genomics in *C. albicans* (17). This work, alongside a paper published by our group (18), provided genomic evidence for mating between lineages in this nonmeiotic species that contribute to the species population structure. Furthermore, the parental lineages for recombinant strains could be identified among the sequenced strains by Ropars et al. (17), to connect putative parental and progeny genotypes. These efforts laid the groundwork to include diverse isolates into molecular approaches dissecting commensalism and virulence.

Here, we describe recent work published mostly in the last 5 years that has expanded our understanding of genome evolution and adaptation in *C. albicans* through (i) use of mutational studies in defined strain backgrounds or (ii) naturally occurring genetic variation among isolates. Use of the genome reference strain SC5314 in evolutionary studies has facilitated an emerging description of *C. albicans* adaptation, whereas use of clinical isolates to dissect how standing genetic variation contributes differences in host interactions is still in its infancy. We note that we are focusing on studies that go beyond accumulating more *C. albicans* genome sequences. Instead, these studies align with the spirit of the two influential papers noted above by linking *de novo* adaptive mutations or existing strain variation to cellular and molecular functions that distinguish the strains and can promote organismal survival or fitness.

## MITOTIC MECHANISMS OF *C. ALBICANS* EVOLUTION

Studies of *C. albicans* evolution focus primarily on mitotic mutational processes that exist in three main groups: point mutations, loss of heterozygosity, and changes in copy number or ploidy. First, point mutations affect a single base pair position and include single nucleotide variants and insertions/deletions (indels) of one to a few nucleotides. The presence of two homologous chromosomes in diploid genomes allows point mutations to be heterozygous or homozygous in *C. albicans* with the exception of hemizygous loci, such as the mating type-like (*MTL*) locus (19). Second, loss of heterozygosity (LOH) can occur through the loss of allelic information on one chromosome homolog and replacement with information from the other homolog. LOH is often a consequence of DNA double-strand break repair and results in homozygosis of heterozygous positions. The length of LOH tracts can extend up to and include an entire chromosome, which occurs as a result of chromosomal nondisjunction and reduplication during mitosis (11). Third, large-scale changes in copy number, such as ploidy or aneuploidy, via chromosomal nondisjunction arise frequently in *C. albicans* strains (12, 20). While full ploidy changes often require either nuclear fusion or complete mitotic collapse to alter the copy number of all chromosomes simultaneously, aneuploidy describes the loss or gain of an incomplete complement of chromosomes that may occur through more subtle errors in chromosome segregation or cytokinesis.

### Experimental and computational approaches expand the role of point mutations in *C. albicans* evolution

Characterization of point mutations in cells with aberrant phenotypes can be used to readily interpret genotype-phenotype relationships. Most studies of *C. albicans* use reverse genetics to target specific genes with designed mutations and not forward genetic screens of randomly produced mutants. Thus, point mutations are generally engineered to explicitly test aspects of gene function in connection to a preconceived phenotype. This bias toward reverse genetics stems, in part, from the challenge in producing loss of function mutations in a diploid species, which require both alleles to be disrupted. To date, genes involved in antifungal drug resistance are most commonly targeted for mutation because of the wealth of mutational data for known drug targets obtained from clinical isolates with defined resistance, the ease of phenotyping through standardized assays, and the relevance to clinical practice (21–25). However, these approaches are being increasingly applied to other phenotypic traits. Studies investigating the yeast-hyphal transition in response to serum (26) and upon engulfment by macrophage (27) used point mutants to dissect cell signaling and phenotypic responses. A major challenge in interpreting point mutants is the need for accurate structure-function predictions. Protein structures have been historically challenging to solve, especially for lineage-specific homologs, but are now greatly aided by artificial intelligence-driven protein folding (28).

Use of experimental evolution, a process by which selection acts on naturally occurring mutations in a population, is ideally suited for microbial populations such as *C. albicans* and has facilitated the identification of genes important to *C. albicans* survival in the host. Additionally, experimental evolution has allowed for interrogation of how the *C. albicans* genome evolves through mutation. Recent experimental evolution work by Ene et al. (2) provided the best current estimates of point mutation rates in *C. albicans* via repeated passaging *in vitro* in rich liquid medium and *in vivo* through the murine gut and bloodstream. Single nucleotide polymorphisms (SNPs) arose *in vitro* at a rate of 1.17 × 10^−10^, which is similar to other fungal organisms, and showed strong biases toward repetitive regions of the genome presumably due to increased error rates during DNA replication and relaxed selection for their removal when they arise. A second study found similar mutational biases toward repetitive sequences and away from coding sequences during *in vitro* evolution of strains with elevated echinocandin resistance (29). In contrast to these *in vitro* studies, mutation rates *in vivo* cannot be evaluated because of the inability to determine generation times. Yet, standardizing mutational frequencies to time suggested higher rates of mutation occur in the gut and bloodstream of infected mice compared with *in vitro* culturing but are limited by being estimates (2).

A major challenge in experimental evolution studies with *C. albicans* is the potential for accumulation of hundreds or thousands of new point mutations in addition to large-scale chromosomal events that can complicate identification of causative loci (2, 29–31). However, one recent study that sought to understand the genetic basis of gut colonization of mice by *C. albicans* was able to identify the genotypic basis of major phenotypic changes *in vivo*. Repeated passage through the murine gut in Tso et al. (4) identified multiple lineages with loss of function mutations in *FLO8* that increased fitness in the murine GI tract. Use of multiple lineages to find a commonly disrupted locus was a key component to the success of the study. Furthermore, this work highlighted the importance of experimental design in evolutionary studies because of its ability to profoundly influence evolutionary dynamics and mutational load in the evolved population (32), which will affect the ease of identifying causative mutations.

Indels are point mutations that often change the length of DNA sequences and are both a product of and contributor to repetitive sequences in genomes. Large repeats in the *C. albicans* genome, such as the ribosomal DNA (rDNA) locus and the major repeat sequence (MRS) found on all but one chromosome, have been used for decades to delineate “sequence types” of genetically similar strains because of their propensity to accumulate indels over evolutionary time (33, 34). The concept of what defines a repeat in the *C. albicans* genome was recently expanded. A focused effort by Todd et al. (35) found ~2,000 cryptic repetitive sequences in the *C. albicans* reference genome, defined as being at least 65 base pairs in length and having at least 80% identity to another locus in the genome. Importantly, these repeats can serve as breakpoints for loss of heterozygosity and copy number variation that contribute to adaptation in the presence of antifungal drugs (35, 36) and in mutant genotypes with decreased fitness (37). The ubiquity of these repeats across the genome coupled with their high sequence similarity may allow homologous recombination to rapidly generate novel genotypes and karyotypes that can overcome various stressors compared with point mutations (38).

Relatively few phenotypes of interest have been linked to indel formation in *C. albicans*. This is likely a result, in part, of selection against frameshift-causing indel formation in protein-coding genes that often reduce organismal fitness (2). However, this bias against insertions in genes required for organismal fitness can also be used to identify loci essential for life using transposon-based insertional libraries. Isolation of a karyotypically stable haploid variant of *C. albicans* facilitated a survey of essential genes and genic domains in the SC5314 genetic background (39). The authors of this study then compared the essential genes identified in *C. albicans*, *Saccharomyces cerevisiae*, and *Schizosaccharomyces pombe* Tn-Seq experiments. Components of the mitochondria and outer kinetochore DASH complex were uniquely necessary in *C. albicans*, which may reflect genetic rewiring and novel structural assemblies in species of the CUG paraphyletic group, respectively. Comparative genomics of fungal orthologs can also reveal recently acquired indels in genes that may shape molecular function. For example, three insertions are present in CUG species orthologs of a predicted squalene/phytoene synthase, named *NDU1*, in *C. albicans* (40). Disruption of either of two adjacent inserts in *NDU1* phenocopied the *ndu1*Δ/Δ mutant, suggesting that these evolutionarily recent inserts in an ancient gene are required for gene function. Alignment of orthologous gene clusters across the *Ascomycota* found recently acquired insertions were present throughout CUG species and may similarly contribute to gene function in this species lineage as was shown for *NDU1* (41). Therefore, studying the evolutionary patterns and consequences of point mutations across large evolutionary timeframes can complement the use of experimental approaches in revealing gene function.

### Short loss of heterozygosity tracts alter complex *C. albicans* phenotypes

Loss of heterozygosity was demonstrated to be an important and clinically relevant mechanism of *C. albicans* adaptation to antifungal drugs approximately two decades ago (42). This early work had binned LOH events based on tract length into whole chromosome, long tract (typically at least a chromosome arm), or short tract (encompassing one of four markers spaced along a chromosome homolog) events (11). Yet, only with adoption of whole-genome sequencing were the frequency and full distribution of LOH tract lengths recently resolved. Experimental evolution of *C. albicans* uncovered the frequent production of “micro-LOH” (mLOH) tracts that occur over short segments of DNA, sometimes only spanning a few nucleotides (2, 43). Production of short homozygous tracts expands the role of recombination in shaping evolutionary trajectories; key beneficial variants can be homozygosed during adaptation without including nearby bystander variants that may decrease fitness. The evolutionary importance of short-tract LOH is demonstrated by the inability to obtain whole-chromosome LOH of certain chromosome homologs in *C. albicans,* presumably due to the presence of lethal recessive alleles (44). Importantly, short LOH segments can shape host colonization dynamics. Homozygosis of *EFG1*-disrupting mutations by small LOH tracts facilitated formation of the gray cell state in multiple *C. albicans* backgrounds, which colonize the murine gut more effectively than their white cell counterparts (45). Like point mutations, the frequency of LOH events increases during host colonization compared with *in vitro* passage and may provide the evolutionary fodder for selection of fit genotypes *in vivo* without requiring *de novo* point mutations (46).

### Aneuploidy interacts with the environment to improve fitness

Changes in chromosome copy number offer an adaptive mechanism without requiring stably inherited mutations or allelic loss via LOH in the *C. albicans* genome. As with LOH, initial functional ties to aneuploidy centered on antifungal drug resistance (47). Continued investigation of the relationship between aneuploidy and antifungal drug responses has recently shown that Chr5 monosomy increases tolerance to the echinocandin drug caspofungin (48), Chr2 trisomy confers caspofungin tolerance via tunicamycin-induced endoplasmic reticulum stress (49), and subinhibitory levels of the azole-class antifungal drug fluconazole can induce widespread aneuploidy (50), presumably by decoupling nuclear segregation and cytokinesis (51). New data also suggest a relationship exists between ChrR trisomy and azole drug tolerance, the ability to grow slowly in the presence of drug, in the genome reference strain SC5314 across a range of fluconazole concentrations (31). Interestingly, fluconazole-tolerant isolates could either contain a full ChrR trisomy, a segmental trisomy that excluded the right arm of ChrR past the rDNA locus, or a segmental monosomy of the right arm of ChrR past the rDNA locus. This suggests that dosage of multiple ChrR loci contributes to azole tolerance or imbalance of ChrR loci alone is sufficient to improve drug tolerance.

Moving beyond simply associative studies of antifungal drugs and aneuploidy, new work has dissected how ploidy contributes to antifungal drug survival. For example, tetraploid *C. albicans* cells more quickly evolved resistance to caspofungin than diploid cells in the same genetic background (52, 53). These tetraploid cells experienced increased genome instability compared with their diploid counterpart as might be expected with more chromosomes, but the reason why remains unclear. More rapid development of antifungal resistance in tetraploid cells could be a consequence of increased DNA copies that can acquire a specific beneficial mutation, increased acquisition of other beneficial mutations that offset fitness losses during antifungal drug adaptation, and/or the increased efficiency of losing chromosomes that do not contribute to resistance versus gain of additional chromosomes by diploid cells that can improve fitness. The accelerated emergence of antifungal resistance in tetraploid cells could also be linked to their reduced growth rates under most conditions; *C. albicans* strains with lower initial fitness in subinhibitory antifungal drug concentrations showed greater improvements in drug resistance than more initially resistant strains, whereas a more complex relationship emerged for drug tolerance (54). Lower initial fitness was linked to production of a larger range of genome sizes, antifungal drug tolerance phenotypes, and fitness phenotypes following repeated passaging in low concentrations of antifungal drug compared with populations with higher initial fitness. Thus, larger fitness deficits of the original genotype may increase the emergence of complex karyotypic variants to access beneficial genotypes. Importantly, recent work has shown that the karyotypic changes selected for by one antifungal drug could threaten the effectiveness of other antifungal agents. Emergence of aneuploidy that confers tolerance to one antifungal drug can facilitate cross-tolerance to other compounds with similar modes of action or through the linkage of genes that mitigate other compounds (55, 56). Taken together, this suggests that the evolutionary path taken by *C. albicans* in the presence of antifungal drugs will not be the same across strains and can be affected by subtle point mutations and environmental conditions that influence the baseline fitness of each strain.

The consequences of aneuploidy have more recently been demonstrated to affect other aspects of *C. albicans* biology and phenotypic diversity beyond the response to antifungal drugs. Feng et al. (57) performed a systematic study of the phenotypic effects of single-chromosome trisomies for either the A or B homolog in the SC5314 background. The only shared phenotype among the 16 aneuploid strains (8 Chr × 2 homologs as trisomic) was a reduced growth rate under *in vitro-*ich medium conditions. Instead, trisomy of individual chromosomes interacted with specific conditions or exposure to unique stressors to alter fitness or survival. Furthermore, the identity of the trisomic homologs (A or B) usually did not change the associated phenotype, suggesting allelic variation had less impact than gene dosage.

Improved *in vivo* fitness of aneuploid strains has also been shown to be closely tied to trisomy of specific chromosomes. Cells harboring a Chr5 or Chr6 trisomy were enriched from an initially diploid SC5314 inoculum in the oral cavity of mice (46). These aneuploid isolates caused less inflammation and tissue damage during oral colonization compared with diploid strains, which may be responsible for their improved fitness in this niche (15). In contrast to the oral cavity, a Chr7 trisomy dominated the recovered population from an initially diploid SC5314 inoculum during gut colonization of mice (2). Follow-up experiments directly competing diploid and Chr7 trisomic strains from multiple genetic backgrounds demonstrated that the aneuploid cells had improved fitness in colonization of the murine gut as well as various organs during systemic infection (3). The increased copy number and expression of *NRG1* on Chr7, which functions as a negative regulator of filamentation, was responsible for the improved gut fitness in the aneuploid SC5314 strain. Interestingly, the *in vivo* environment itself may increase production or retention of aneuploid cells as was recently demonstrated in nematode models of infection, but this increase in aneuploidy was not induced by host immunity (58, 59). Thus, some other aspects of the host niche environment may increase aneuploid frequency that selection can then use to enrich for fitter karyotypes.

The coordination of nuclear replication and cell division is required to maintain genome integrity across cellular organisms, but relatively few *C. albicans* genes are known to explicitly contribute to genome stability. Identification of a role for the SUMO-deconjugating enzyme *ULP2* in promoting genome stability may provide some interesting insights into how this operates in *C. albicans* (37). Strains lacking *ULP2* displayed abnormal chromosomal segregation and formed segmental aneuploidies at high frequency. The molecular targets for SUMOylation that are required for genome integrity are not known, but it is assumed that these proteins are likely involved in cytokinesis, nuclear division, or cell signaling to coordinate these processes.

## MATING AND PARASEX HAVE INFLUENCED *C. ALBICANS* GENOME EVOLUTION

Although no meiotic program has been observed in *C. albicans*, an alternate mating program termed parasex has been characterized for this and closely related *Candida* species (60). As opposed to the ordered halving of DNA that occurs during production of meiotic products that will fuse with another germ cell to restore the original ploidy of the organism, parasex is highly uncoordinated and often results in highly aneuploid progeny. In *C. albicans*, an epigenetic conversion from the “sterile” white cell state to the mating competent opaque state is often required for mating competence. Heterozygosity of the idiomorphic *MTL* locus blocks transition to the opaque state under many conditions, and this locus must undergo homozygosis or loss of one of two alleles to undergo white-opaque phenotypic switching. Once in the opaque state, cells of opposing mating types secrete and respond to pheromone from cells of the alternative mating type and migrate toward one another through a polarized cell extension or “schmoo”. Cytoplasmic and nuclear fusion occurs on contact of two cells of opposing mating types to produce a mating product that contains the complete genetic material from both parental cells. This mating product can grow mitotically or can be induced to enter parasex, an uncoordinated process of ploidy reduction. Parasex is most efficiently induced when tetraploid mating products are grown on high sugar pre-sporulation (pre-spo) agar medium originally developed to assist poorly sporulating *Saccharomyces cerevisiae* strains (61). Parasexual progeny often fails to return to diploid DNA content, remaining aneuploid, and can contain a mixture of parental genotypes.

Mechanistic aspects of parasex are severely understudied compared with meiosis, but key insights have been aided by recent molecular analysis. Transcriptional profiling of tetraploid cells grown on pre-spo medium to instigate parasex revealed signatures of DNA damage production that were associated with a strong oxidative burst resulting from growth of *C. albicans* tetraploid cells on the pre-spo medium specifically (62). The introduction of DNA double-strand breaks during parasex was verified by expression of a viral GAM protein in *C. albicans*, which binds double-strand DNA breaks and strongly labeled *C. albicans* cells undergoing parasex (63, 64). Furthermore, this labeling was partially dependent on the “meiosis-specific” nuclease *SPO11*, suggesting both environmental (e.g., ROS) and encoded (e.g., Spo11) sources of DNA breaks contribute to recombination. The meiotic cohesin Rec8 is also important for ploidy reduction and recombination during *C. albicans* parasex (63), suggesting that many meiotic proteins may have been co-opted for parasex.

Sequence analysis is providing insight into how genetically diverse lineages of *C. albicans* are produced by parasex. As opposed to the controlled halving of DNA during meiosis that restores the ploidy of the parental cells in meiotic products, parasexual mating products in *C. albicans* are overwhelmingly aneuploid (63, 64). Generation of a highly aneuploid pool of parasexual progeny would increase the genetic diversity of resident populations that may facilitate the emergence of new phenotypes and improved fitness. In addition, frequent recombination between homologs during parasex would add to the genetic diversity of progeny by producing new allelic combinations. Early estimates of parasexual recombination using comparative genomic hybridization of single nucleotide polymorphisms (SNP/CGH) detected only low levels of recombination, although this may have been an underestimate since the parental genotypes were nearly identical and genotyping markers were separated by 92 kilobases of DNA, on average (64). More recent estimates of recombination during parasex used a tetraploid SC5314xSC5314 mating product with selectable markers phased on a single Chr1 homolog spaced either 50 or 400 kilobases apart (63). Unexpectedly, recombination rates between these marker sets were similar, suggestive of high levels of recombination during parasex that may be instigated by DNA damage from free oxygen radicals when grown on pre-spo medium (62). Yet, the phenotypic diversity produced by parasex is just beginning to be explored as it has only been investigated in progeny from mating isogenic strains (65). Nevertheless, parasex between fluconazole-resistant parental lineages with distinct mutations can produce highly resistant progeny that contain both parental mutations and highlights the potential for this process to facilitate adaption via DNA exchange between divergent *C. albicans* lineages (66).

Sequencing diverse clinical isolate genomes supported a role for parasex in generating multiple distinct *C. albicans* lineages. These recombinant strains appeared in each of the two large, sequenced sets of clinical isolates, and the parental lineages involved in each mating and parasexual events could be inferred from their genome composition (17, 18). Interestingly, both of the two recombinant strains among a set of 21 sequenced isolates were highly resistant to azole antifungal drugs and may suggest a relationship between mating and azole drug exposure, which can induce genome rearrangements and *MTL* homozygosis (51).

Mating between diverse lineages or distinct species without ploidy reduction can lead to hybrid species that are distinct from either parental genotype. Analysis of CUG paraphyletic group genomes suggests ancient hybridization events between fungal species may have given rise to multiple members of this species cluster, including *C. albicans* (67). The sequence of homologous chromosomes in *C. albicans* is quite divergent, encoding a heterozygous position every ~204 base pairs on average (17). In comparison, *S. cerevisiae* and *C. parapsilosis* encode a heterozygous position approximately every 2,000 base pairs on average each (68, 69). These heterozygous positions lie in blocks, interspersed by regions of LOH. Analysis of heterozygous positions across phylogenetically diverse *C. albicans* strains found that most variants were identical and that assignments of variants to each chromosome homolog resulted in two haplotypes that were more divergent from each other than between different strains (67). This result is consistent with evidence of hybridization in other *Saccharomycotina* species, including multiple other *Candida* species (70). However, the identity of the parental lineages that led to the hybrid *C. albicans* genome remains elusive.

## INCREASED USE OF DIVERSE STRAINS TO STUDY *C. ALBICANS* EVOLUTION

**Fig 1:**
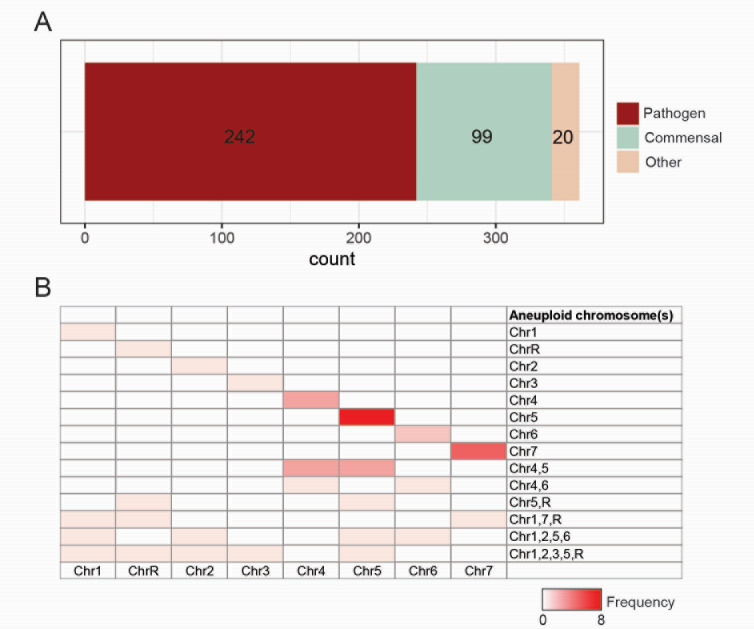
Features of natural isolates used in molecular investigations. (**A**) The origin of isolates listed in Table 1 was determined and plotted. Other indicates isolates from either food sources or unknown origins. (**B**) The frequency of aneuploid karyotypes for different chromosomes is plotted for the 361 natural isolates. The heat map indicates the frequency of the given karyotype.

The reduced cost of short-read (e.g., Illumina) and long-read (e.g., Oxford Nanopore) sequencing technologies and standardization of analytical pipelines for genome analysis (for details see references 71–73) have allowed researchers to access a wide breadth of *C. albicans* genetic diversity. Currently, the genomes of hundreds of commensal and clinical isolates are available through deposits to various genome repositories (e.g., Sequence Read Archive, European Nucleotide Archive, and DNA Data Bank of Japan). Among these, two main strain sets have emerged for use in computational and experimental investigations: the 21 strains sequenced as part of the Hirakawa et al. study (10)and the 182 strains from the Ropars et al. study (17). The 21-strain set is comprised of clinical isolates collected from the bloodstream, in part, through the SENTRY Surveillance program in the United States (74), with some initial characterization by David Soll’s lab (75). In contrast, strains from Ropars et al. were isolated primarily from either superficial infections or as commensals from the gastrointestinal tract or vagina of their healthy human hosts. Differences in the host condition and niche of isolation between these and other strains could provide continued insight into comparing commensal and pathogen genotypes and behaviors. To accomplish this, the field should heed calls for more commensal isolates to be recovered from healthy individuals to better understand the selective pressures that contribute to *C. albicans* adaptation as a common member of the human microbiome (Fig. 1) (76). Since most clinical infections stem from a commensal source (77), being able to tease out any characteristics that increase the likelihood of a strain to cause disease requires a better understanding of commensal diversity and how commensal populations may be shaped by the transition to mucosal or systemic disease. This is particularly important given that many recent studies have indicated that the SC5314 genome reference strain and its derived auxotrophic lineages poorly represent the species’ responses and phenotypes in many cases (78–81).

**TABLE 1:** Studies using diverse *C. albicans* strain backgrounds

Study	Focal topic	Isolate included	Reference
Ene et al.	Microevolution	SC5314, P78048, P76055, and P57055	2
Butler et al.	Genome evolution	SC5314, WO-1	8
Hirakawa et al.	Intra-species variation	SC5314, GC75, P75016, P75063, P87, P60002, P94015, P34048, P78042, P57055, P57072, P76055, P76067, P75010, 19F, L26, P37039, 12C, P37005, P37037, and P78048	10
Ford et al.	Genetic variation during clinical infection	TWTC1-17, Perea et al. isolates (82)	13
Ropars et al.	Intra-species variation	SC5314 and 181 isolates (117 clinical, 45 commensal, and 19 food derived)	17
Zuber et al.	Antifungal resistance	SC5314, JRCT1	29
Yang et al.	Antifungal resistance	SC5314, T490, and T1891	31
Todd et al.	Genome plasticity	SC5314, P78042	35
Todd and Selmecki	Antifungal resistance	SC5314, P75016, P75063, and P78042	36
Todd et al.	Genome plasticity	SC5314, P75063, and FH1	38
Coste et al.	Antifungal resistance	SC5314, T118, DSY347, DSY731, DSY2321, and DSY3534	42
Liang et al.	Commensal-pathogen balance	Twenty-one isolates from Hirakawa et al. (5), 43 isolates from Ford et al. (8), BJ1097, HJ039, and HJ071	45
Yang et al.	Antifungal resistance	SC5314, JRCT1	48
Gerstein and Berman	Antifungal resistance	AM2003.089, AM2003.0165, AM2003.0069, DSY294, FH1, OKP90, T118, T101, and 21 isolates from Hirakawa et al. (5)	54
Kukurudz et al.	Antifungal resistance	SC5314, FH1, GC75, P75016, P76055, P78048, P87, and T101	55
Smith and Hickman	Genome plasticity	SC5314, FH1, FH6, PN1, and PN2	59
Hirakawa et al.	Parasexual diversity	SC5314, 529L, HUN92, J981315, L1086, P60002, and YSU751	65
Wu et al.	Virulence phenotypes	Twenty-one isolates from Hirakawa et al. (5)	75
Anderson et al.	Genetic variation during commensalism	SC5314, CHN1, and 910 commensal isolates	78
Dunn et al.	Virulence phenotypes	Twenty-one isolates from Hirakawa et al. (5), 529L, and WO-1	79
Glazier et al.	Transcriptional regulation	SC5314, P57055, P75010, P76067, P87, and RO39	80
Brandquist et al.	Filamentation	Twenty-one isolates from Hirakawa et al. (5), 20 clinical isolates	81
Sitterle et al.	Antifungal resistance	One hundred and fifty-one isolates from Ropars et al. (11), 10 azole-resistant clinical isolates	83
Sitterle et al.	Genetic variation during commensalism	Forty-nine commensal isolates	84
Moorhouse et al.	Genetic variation during commensalism	Three hundred sixty-nine commensal isolates	85
Gnaien et al.	CF colonization and disease	Eighteen CF isolates (HBJ#-#)	86
Kim et al.	CF colonization and disease	Three CF isolates (F1, Y1, and Y2)	87
Wang et al.	Virulence phenotypes	Twenty-one isolates from Hirakawa et al. (5)	88
Huang et al.	Biofilm formation	SC5314, P57055, P75010, P76067, and P87	89
Cravener et al.	Biofilm	SC5314, 12C, 19F, GC75, L26, P37005, P37037, P37039, P57055, P57072, P75010, P75016, P75063, P76067, P78042, P78048, and P87	90
Mao et al.	Filamentation	SC5314, P57055, P75010, P76067, and P87	91
Liu et al.	Strain diversity	SC5314, 529L	92
McDonough et al.	Commensalism	SC5314, 529L, and CHN1	93
Kosmala et al.	Commensal-pathogen balance	SC5314, 529L, and 35 *C*. *africana* isolates	94
Millet et al.	Commensalism	SC5314, 529L, and CA101	95
Lemberg et al.	Oral colonization	SC5314, 101, 529L, Cag, CEC3609, CEC3617, CEC3621, CEC3672, and CEC3678	96
Sala et al.	RVVC	SC5314, 22 vulvovaginal candidiasis isolates, and 13 commensal isolates	97
Roselletti et al.	VVC	CA-67, CA-105	98
Li et al.	Inflammatory bowel disease	SC5314, ID(A/B/C/D)###	99

Two main strategies for use of diverse *C. albicans* isolates have been pursued by research groups thus far. The first approach begins with sequenced genomes to identify variants that can be tested for phenotypic relationships. For example, mutations in genes known to promote azole and echinocandin antifungal drug resistance were catalogued among the drug-susceptible Ropars strain set (83). These mutations could then be screened against drug-resistant isolates and shared variants could be marked as potential resistance mutations. Comparison to drug-susceptible natural isolates facilitated construction of a more focused list of variants of interest for investigation in known drug resistance genes. However, this approach requires focusing on genes that are explicit drug targets or unlikely to interact with other proteins to confer phenotypes. In general, expanding comparative genomics approaches to distantly related strains is complicated by the enormous genetic diversity found in *C. albicans*. Instead, use of closely related isolates can expedite identification of diverse genotype-phenotype relationships. This is facilitated by increasing evidence showing that colonizing *C. albicans* populations are often genetically heterogeneous in single individuals and can serve as an ideal source for genotypically distinct but related strains harboring a small number of unique mutations (78, 84, 85). Indeed, Anderson et al. (78) isolated 910 *C*. *albicans* colonies from colonized niches of 35 healthy adults and performed whole-genome sequencing and deep phenotyping on a large subset of the recovered isolates. Comparison of genetically related colonies derived from a single individual found a heterozygous mutation in the *ZMS1* transcription factor associated with substantially greater substrate invasion *in vitro*. Introduction of this heterozygous variant into a related non-invasive strain or removal of this variant from the invasive strain flipped the invasion phenotype. Similar approaches have led to the identification of heterozygous variants in the *ROB1* and *NRG1* transcription factors that govern filamentation and biofilm formation in strains recovered from cystic fibrosis (CF) patients (86, 87). Moving past DNA, transcriptional profiling of the 21-strain set was able to cluster previously uncharacterized genes into modules of predicted related functions that were found to be accurate when tested experimentally (88). These unbiased approaches require significant amounts of data to produce testable hypotheses but have repeatedly produced new understandings of gene function among *C. albicans* isolates.

The other major strategy for investigating clinical isolates begins by characterizing phenotypic differences that can be linked to causative genetic variants using available sequencing data. While many studies have phenotyped strains broadly, most have not taken the steps to link these differences to genetic loci. Huang et al. conducted some of the first phenotype-to-genotype investigations to connect differences in biofilm production by the 21-strain set to variation in connectivity among the interlocking transcriptional network that controls this phenotype in SC5314 (89). This work showed that even well-characterized transcription factors in *C. albicans* do not hold identical functions across strain backgrounds and was well supported by subsequent studies of other transcriptional regulators in diverse strains (90, 91).

Strains that colonize the host without causing significant pathology (e.g., 529L, CHN1, and isolate 101) have also been a focal point of recent investigations into clinical isolates (92–98). The genetic basis for many of these differences in colonization, immune responses, and gross pathology are not known. However, investigation of 529L showed that sequence variation in *ECE1*, which encodes the fungal toxin candidalysin, is partly responsible for its reduced virulence and inflammation compared with SC5314 (92). Variants at the Kex2 cleavage site between *ECE1* peptides 2 and 3 (candidalysin) in 529L likely reduce proper processing of the toxin despite high expression of the *ECE1* RNA. Loss of *ECE1* in SC5314 and clinical isolates introduced into the murine gut also reduced the Th17 response and colonic neutrophil infiltration but did not inhibit filamentation, linking immune pathology to toxin production and not morphology (99). The genetic regulators of other commensal-like behaviors in these strains, including reduced hyphal formation in the presence of host cells, that may contribute to the commensal-pathogen balance, remain unidentified.

## CONCLUDING REMARKS

The reduction in sequencing costs combined with the increasing availability of diverse isolates has accelerated investigations of genetic variation in *C. albicans*. The major steps taken to include diverse strains in *C. albicans* research is promising, and inclusion of additional isolates is likely to yield continued insights into fungal evolution and adaptive mechanisms. Yet, these studies will need to be designed intentionally to maximize what can be learned beyond the previously described patterns of genomic change. Connecting genotypic changes that alter phenotypic responses will likely be a fruitful approach to define gene function for a large portion of the *C. albicans* genome that remains uncharacterized. Furthermore, continued broad genotype-phenotype investigations may provide insight as to whether all *C. albicans* strains are adept commensals with the ability to cause opportunistic disease or if some strains are better adapted to be commensals or pathogens in the human host. This work will benefit from both evolution of well-characterized strains and comparative genomics of clinical isolates. The *C. albicans* species holds an enormous wealth of genetic diversity; we as a community are just starting to scratch the surface.
